# The Learning Exchange, a Community Knowledge Commons for Learning Networks: Qualitative Evaluation to Test Acceptability, Feasibility, and Utility

**DOI:** 10.2196/formative.9858

**Published:** 2019-03-14

**Authors:** Daniel McLinden, Sarah Myers, Michael Seid, Melida Busch, David Davis, John Murphy

**Affiliations:** 1 Department of Pediatrics College of Medicine University of Cincinnati Cincinnati, OH United States; 2 Idea Networks Covington, KY United States; 3 James M Anderson Center for Health Systems Excellence Cincinnati Children's Hospital Medical Center Cincinnati, OH United States; 4 Division of Pulmonary Medicine Cincinnati Children's Hospital Medical Center Cincinnati, OH United States; 5 Edward L Pratt Research Library Cincinnati Children's Hospital Medical Center Cincinnati, OH United States; 6 Learning Sciences Cincinnati Children's Hospital Medical Center Cincinnati, OH United States; 7 Evaluation, Research and Measurement Cincinnati Children's Hospital Medical Center Cincinnati, OH United States

**Keywords:** quality improvement, knowledge management, community networks, intersectoral collaboration, database management systems, patient-centered care

## Abstract

**Background:**

Learning Networks are distributed learning health systems that enable collaboration at scale to improve health and health care. A key requirement for such networks is having a way to create and share information and knowledge in furtherance of the work of the community.

**Objective:**

We describe a Learning Exchange—a bespoke, scalable knowledge management and exchange platform initially built and tested for improving pediatric inflammatory bowel disease outcomes in the ImproveCareNow (ICN) Network—and assess evidence of its acceptability, feasibility, and utility in facilitating creation and sharing of information in furtherance of the work of the community and as a model for other communities.

**Methods:**

Acceptability was assessed via growth in active users and activity. Feasibility was measured in terms of the percentage of users with a log-in who became active users as well as user surveys and a case study. Utility was measured in terms of the type of work that the Learning Exchange facilitated for the community.

**Results:**

The ICNExchange has over 1000 users and supported sharing of resources across all care centers in ICN. Users reported that the Learning Exchange has facilitated their work and resulted in increased ability to find resources relevant to local information needs.

**Conclusions:**

The ICNExchange is acceptable, feasible, and useful as a knowledge management and exchange platform in service of the work of ICN. Experience with the ICNExchange suggests that the design principles are extensible to other chronic care Learning Networks.

## Introduction

### Background

In the US health care system, patients receive only 50% of recommended care [1,2] and only 50% of those patients are able or have the necessary support to follow the recommendations [3]. Mindful of this, the Institute of Medicine (now the National Academy of Medicine) has called for learning health systems [4] in which patients and clinicians work together to choose care based on best evidence and drive discovery as a natural outgrowth of every clinical encounter, ensuring innovation, quality, and value at the point of care. Learning Networks [5]—organizational structures that facilitate coproduction [6] to improve health and health care—are promising examples of such learning health systems [7]. However, to reach their potential, Learning Networks must be able to leverage the collective intelligence of large groups of stakeholders—patients, families, clinicians, and researchers—to distribute both the production and implementation of information, knowledge, and know-how [8].

Fjeldstad and colleagues [9] have described an organizational architecture that might enable large-scale coproduction in systems like Learning Networks. This actor-oriented architecture consists of (1) sufficient numbers of actors (people and organizations) with the values and capabilities to self-organize, (2) structures, protocols, and processes that make it easy for actors to form highly functional teams, and (3) a commons where actors create and share information, knowledge, and know-how. A recent scoping review of collaborative writing applications such as wikis suggests that such knowledge translation platforms are in increasing use [10], although evidence for their impact is still lacking [11]. However, the Learning Networks previously had no purpose-built knowledge commons platform that could engage large numbers of diverse stakeholders.

Below, we describe the design and use of such a platform, the Learning Exchange, within the setting of ImproveCareNow (ICN), a Learning Network whose mission is to transform the health, care, and costs for children and adolescents with inflammatory bowel disease (IBD) by enabling patients, families, clinicians, and researchers to work together to accelerate innovation, discovery, and the application of new knowledge [12,13]. When it formed in 2007, ICN consisted of 8 care centers from 7 states. Currently, more than 900 pediatric gastroenterologists from more than 100 ICN care centers in the United States, United Kingdom, Qatar, and Belgium care for more than 28,000 children with IBD. During this decade, the remission rate for patients cared for across the Network has increased from approximately 50% to 81%, 96% of patients do not take steroids, and 93% have satisfactory growth status [14].

ImproveCareNow uses the evidence-based chronic care model [15-19] as the framework for improving care. Participating care centers receive instruction and ongoing quality improvement (QI) coaching to build skills and capacity [12,13]. Clinicians enter data for patient encounters into their institution’s electronic health record, from which it is then passed into the ICN2 registry where it populates measures and reports that drive care decisions [20]. Local QI teams consist of clinicians, QI consultants, researchers, patients, and parents from each center. At monthly teleconferences and semiannual face-to-face meetings based on an adapted breakthrough series (BTS) method [21,22], the teams transparently share best practices, outcome data, and lessons learned from changes they are testing. There is also a robust, asynchronous communication infrastructure featuring a newsletter, blog, and social media platforms on Twitter, Facebook, and Instagram [23]. All ICN members are encouraged to continuously design and test network-wide, center-specific, and personal innovations to make collaborative and participatory care more efficient and effective, ultimately leading to improved outcomes. They also use communication structures to share the status of innovations with the network and learn from and apply the work from other centers to their own. Current QI projects focus on engagement, self-care and care management, and chronic illness care and sustainability.

In the adapted BTS model, care center QI teams, comprised of patients, families, clinicians, QI staff, and researchers, meet in person twice a year and participate in monthly webinars. These are synchronous and high-touch approaches that are vital but not sufficient as methods to scale and meet the needs of a large, growing, and geographically dispersed community. While attendees were aware of what was being done by others, the schedule of regular but infrequent meetings did not provide the structure needed to share information, tools, and ideas robustly throughout the Network or the access those things when the need arose. We needed a place where members of the ICN community could create a shared body of knowledge, tools, and processes and learn from each other about how to improve care and outcomes. We therefore developed the process and technology to create an online community commons, a Learning Exchange [24], and implemented this approach to improve the care for children and adolescents with IBD on a digital platform called the ICNExchange (icnexchange.org). More than a website, a Learning Exchange is community-focused rather than technocentric and can serve as a prototype for other Learning Networks addressing other conditions and a larger network of Networks.

### Design Concept

Based on the principles of open innovation, in 2011 a design team began discussions on the needs of the Network with members of the ICN community. The result was an overall aim to create a learning resource to radically improve what people know about how to implement an effective and reliable care delivery system to treat and manage chronic disease, beginning with pediatric IBD.

The ICNExchange was conceptualized as a visually focused Web platform offering image-cued discovery, curation, and sharing. This choice was inspired by the success of Pinterest (pinterest.com), a platform that experienced substantial growth in a short period of time [25]. We hoped to take advantage of that velocity with a visual-style Learning Exchange. Also, like Pinterest, we envisioned the ICNExchange as a place to share ideas that others can then use or, in the words of the Pinterest CEO, “Our hope is that when we show you the right idea you go out and do that thing”[26].

### Design Activities

Achieving the global aim was conceptualized as delivering on four key drivers: user interaction, technology, content management, and community engagement.

#### Interaction Design

We applied the principles of interaction design [27], including the development of personas and scenarios, to guide our work. Personas are detailed descriptions of users, and scenarios describe tasks that a persona performs with the technology to achieve a goal. The design team built on prior work with personas [28] and developed scenarios that described how a user persona would interact with the technology to achieve a specific goal. For example, a clinician at a care center who wants to track and improve patients’ adherence with their prescribed treatment could log on to the ICNExchange and search for tools and processes implemented by other care centers and create a posting in a forum to which others can respond asking for ideas and resources. The clinician could then download the relevant tools that are attached to the pinned image. These attachments may be documents, spreadsheets, or presentations. Such scenarios, validated with community members, drove the development. In this scenario, the technology must allow search, file attachment, and access to the source files for downloading. In addition, the clinician’s search also requires that content be discoverable. That is, the person who originally shared the resource must have categorized it into a preexisting taxonomy and tagged it with descriptors in their own words.

#### Technology Design

Drupal [29] was chosen as the prototype platform because themed distributions were available to implement the Pinterest-like visual model, a critical design requirement. Additionally, Drupal was open-source software, so the core software was free to use and numerous modules could be licensed to extend capability as user needs and preferences emerged. A key design requirement to encourage widespread use was for content uploading and tagging to be as effortless as possible. The pin motif served this purpose because it was a familiar approach to many members of the community and relatively easy. A pin is a picture that visually signals information. The information may be entirely contained with the image or in addition to the image; the visual may signal that the user has attached files to the pin. Self-contained pins might, for example, signal the availability of a resource at a Web location outside of the ICNExchange, such as a link to a video on YouTube. Pins with attachments might, for example, signal an attached document describing a shared process from a care center. Attached files could be documents, presentations, and worksheets that would be useful for other care centers wishing to implement or adapt that process.

#### Content Management

To be useful, content needs to be readily accessed through a variety of end-user navigational behaviors such as browsing (through visual cues) or searching by descriptors, tags, or categories. One approach is to create and apply taxonomic structure. Taxonomy is critical to ensuring a shared understanding of the organization of content contributed across a diverse community. In this case, content was initially organized into categories that reflected the chronic care model [15-19]: population management, previsit planning, self-management, and data quality. In order to pin an item, the user was required to assign a taxonomic category. The design also allowed users to add their own descriptions or tags to resources to enable different views of content organization to emerge, a folksonomy. A folksonomy is user-generated and emerges from user perspectives about how content should be organized. The “...main advantage is that the [folksonomy] reflects the information structures and relationships that people actually use” [30]. The resulting hybrid taxonomy-folksonomy ontology provided multiple entry points for the user’s discovery process. The tracking of the folksonomy over time also creates the opportunity for the community’s own understanding of content organization to be formalized into a recognized taxonomic structure and navigational cues.

Individual users can curate their own content into boards—collections of resources or pins that a user creates and names (eg, “Good resources for teens” has pins related to self-management and IBD education for adolescents). Users create boards to manage content so that information they have accessed through targeted searching or browsing is readily available. These boards may be followed by other users who wish to learn what someone else finds useful. This feature opens the possibility for some users to demonstrate leadership by curating content and others to follow and observe what these other users find useful.

Content management becomes crucial as the number of resources grows and the network scales. By making resources visible to the care community and providing access to download, repurpose, and readapt content, all users in the community have ready access to the wealth of resources from all care centers. Sharing what exists can be seen as an early phase of a community, a place where shared resources make it easy to search for what you need and browse what is available.

#### Community Engagement

Leadership in online communities is critical to success. Actions by people “...who have the ability to trigger feedback, spark conversations within the community, or even shape the way that other members of a group talk about a topic...” [31] are necessary. To that end, the design team enlisted the commitment of key leaders in the community to actively contribute content, comment on contributions by others, and encourage this same behavior in other members of the community. We identified an activist to lead the community by example by posting pins, commenting on pins, and posting in the discussion forum. The activist was a well-respected member of the community in a leadership role focusing on QI and could call community attention to a particularly valuable or otherwise useful resource; advocate that other users view, download, and adapt a resource; and initiate a chat topic and encourage others to participate. In turn, this leadership was expected to enable and encourage three specific actions in the community: (1) adding resources to the ICNExchange that health care professionals in a care center find useful so these resources are available to the whole ICN community, (2) sharing resources adapted or improved by a care center by pinning to a board so that improved resources are readily available for use in other care centers, and (3) identifying needed resources through collaboration among community members with common interests working across boundaries (role, location, etc) to cocreate them.

### Deployment

We executed a low-fidelity prototype site based on the proposed design. As the prototype was socialized with selected key community leaders, features were implemented or removed based on response. For example, blogging capability initially seemed important but was removed from the ICNExchange because it was incidental to the collaborative information exchange that was evolving in the design. Key features retained included functionality that enabled uploading content, downloading content, and adding remarks such as questions, comments, and suggestions to pins. This latter functionality centered on the pin, allowing the pin to become the focus for new iterations of content and promoting the idea of coproduction within a collaborative community.

Pilot testing was opened to key leaders and selected users, representative of all community roles. Webinar training sessions were provided to give them the knowledge and proficiency to begin using the ICNExchange within their current work processes. In February 2013, five lead innovators, representing a cross-section of network roles and user experience levels (1 beginner, 2 intermediate, 2 experienced), participated in semistructured interviews to gain feedback on site features and develop an understanding of how different users interacted with the ICNExchange. Lead innovators were defined as community members who were likely early adopters and in a position to influence the community regarding adoption and worked closely with the development team to describe needs and test features. Interviews were designed to evaluate the areas of user experience, attitude, and behavior. Data from these interviews were organized according to these areas and synthesized, through comparison, into themes. Usability of the initial prototype was refined and the availability of the ICNExchange was announced with a live demonstration at the spring 2013 ICN Community Conference, an ICN-wide event.

### Ongoing Design

The ICNExchange was not just a new resource; it suggested new ways of working in the community. As such, not all needs could be articulated in the design phase. Users needed to experience the ICNExchange before they could more fully identify needs that could be translated into technology features. Thus, design was considered an ongoing activity. As the community interacted with the commons, their needs emerged through both formal inquiry and informal conversations. These needs were reviewed by the design team and network leadership. Changes were developed and tested in a controlled environment and the revised ICNExchange was released to select users in their real environment before being released to the entire community.

### Study Aim

The aim of this study was to assess the ICNExchange’s acceptability, feasibility, and utility in facilitating the creation and sharing of information in furtherance of the work of the community.

## Methods

### Overview

This project was reviewed by the Institutional Review Board at Cincinnati Children’s Hospital Medical Center and designated as not human subjects research. We used existing data on the number and types of users, their activities, and the content of their posts. We also surveyed users about the ICNExchange and performed a success case evaluation [32].

### Acceptability

We defined acceptability in terms of growth in active users and activity. The Drupal database was queried monthly to determine who was using the platform (ie, role and care center) and what they were doing (eg, adding content as a pin, commenting on a pin, commenting in a forum). In July 2015, we began tracking which files attached to pins were being downloaded by users and examining the popularity of available resources (ie, most downloaded). Activity, including how many people were using the commons, was reported to stakeholders each month to assess whether growth in users was keeping pace with the growth of the Learning Network.

### Feasibility

We differentiated between users, who have an account only, and active users, who have done an action beyond logging in such as viewing a pin or entering a forum at any point in time. We defined feasibility in terms of the percentage of users with a log-in who became active users, as well as data around barriers to use from the user survey and case studies.

### Utility

We defined utility in terms of the type of work that the ICNExchange facilitated for the community. We tracked user actions such as adding to the commons by pinning new resources; repinning an existing resource by, for example, selecting an existing resource and adding it to a personal board; adding comments to a pin, such as by advocating others review this resource; and creating or contributing to discussions in the chat forum. Other analyses examined users by role within the network and resources by type. Of particular interest was determining if other users emerged as leaders by mimicking the actions and activity of the activist.

In September 2013, a cross-section of potential users (n=51) including physicians, coordinators, and parents responded to a survey regarding use, barriers to use, and value. Respondents were also asked open-ended questions about how the ICNExchange has been useful in their work, making the ICNExchange more useful, and areas of additional feedback. Qualitative data from these were organized in lists by a research assistant and one of the authors (SM) and sorted into similar themes, with representative quotes selected to characterize the feedback. Disagreements were resolved by consensus.

In the spring of 2014, a success case evaluation [32], in which in-depth qualitative data are gathered on successful and unsuccessful instances, was implemented with network members from the most (n=10) and least active (n=10) centers. We attempted to contact 35 of the most active Exchange users, mostly nurses or improvement coordinators but some physicians, from these centers via email. Of the 35 individuals contacted, 14 responded with a willingness to participate. We performed semistructured interviews via conference call lasting approximately 30 to 45 minutes with these individuals to determine successful practices and identify recommendations for revisions to improve impact.

## Results

### Acceptability

**Figure 1:**
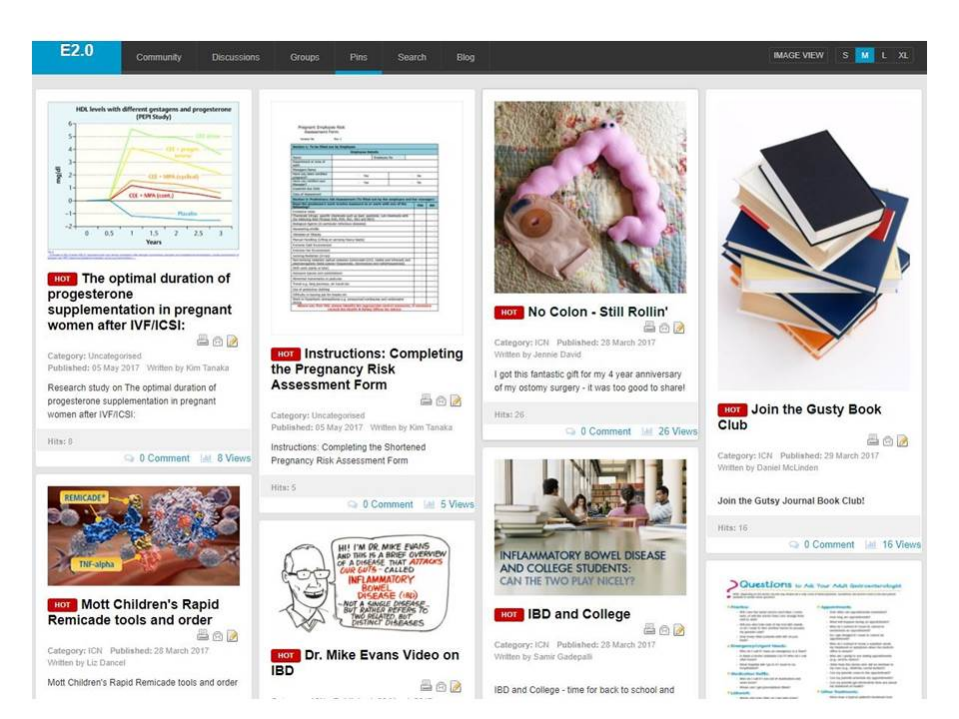
Screenshot of the ICNExchange home page. The screen is dynamic; new resources are added to the top of the page and earlier postings move down.

The ICNExchange (Figure 1) was launched at the April 2013 ICN Community Conference to approximately 200 initial users. By May 2017, there were 1098 users and over 4000 total actions consisting of pins, comments on pins, and posts in the discussion forum. Users worked in a variety of roles (Table 1). The approximately 100 original resources on the Exchange were, for the most part, existing resources taken from other repositories. Since then, the number of contributions has grown, and the content consists of pins and repins, comments on content, and contributions to discussion forums (Table 2).

### Feasibility

While the number of users increased over 5-fold, 72.50% (796/1098) of users became active users. Table 1 shows the percentage of active users by type. As might be expected, improvement coordinators and QI consultants had the highest proportion of active users.

### Utility

The results of the February 2013 interviews with lead innovators in the Network suggested that, while the ICNExchange overwhelmed these early users at first, most felt that the format was engaging, intuitive, and timely. A total of 51 people from 30 care centers responded to the September 2013 survey about use, barriers to use, and value. These included 11 improvement coordinators, 10 nurses, 5 parents, 13 physicians, and 9 other. Overall, 69% (35/51) of respondents had used the ICNExchange. While most improvement coordinators (8/11), nurses (10/10), parents (4/5) and other roles (6/9) indicated that they used the ICNExchange to upload content, download content, participate in a forum, or add a comment, most (7/13) of the physician respondents indicated that they were not likely to use the ICNExchange, citing time constraints (4/13) and usefulness (3/13) as barriers. Of the 35 respondents who used the Exchange, 89% (31/35) agreed that the Exchange is a useful resource for supporting QI activities.

**Table 1:** Breakdown of users of the ICNExchange by role.

User role	Registered users (n=1098), n (%)	Active (n=796), n (%)	Inactive (n=302), n (%)
Physician	264 (24.0)	192 (72.7)	72 (23.8)
Improvement/research coordinator	161 (14.7)	148 (91.9)	13 (4.3)
Parent	157 (14.3)	111 (70.7)	46 (15.2)
Nurse (RN^a^, LPN^b^)	138 (12.6)	96 (69.6)	42 (13.9)
Midlevel practitioner (NP^c^, PA^d^)	61 (5.6)	47 (77.0)	14 (4.6)
Dietician	54 (5.0)	44 (81.5)	10 (3.3)
Patient	39 (3.6)	27 (60.2)	12 (4.0)
Social worker	23 (2.1)	15 (65.2)	8 (2.6)
Psychologist/counselor	17 (1.6)	14 (82.4)	3 (1.0)
Quality improvement coordinator	14 (1.3)	12 (85.7)	2 (0.7)
Project manager	8 (0.7)	5 (62.5)	3 (1.0)
Other (researchers, data architects, hospital administrators, and pharmacists)	162 (14.8)	85 (52.5)	77 (25.5)

^a^RN: registered nurse.

^b^LPN: licensed practical nurse.

^c^NP: nurse practitioner.

^d^PA: physician assistant.

**Table 2:** Breakdown of activities by ICNExchange users.

Resource type	Value (n=4069), n (%)
Adding a resource by pinning content	526 (12.9)
Adding an existing resource to a personal board by repinning existing content	1906 (46.8)
Adding a comment to an existing pin	425 (10.4)
Creating a discussion forum	179 (4.4)
Adding comments to a discussion forum	1033 (25.4)

The 14 case interviews [32] conducted in the spring of 2014 showed that the work done by members from the most active centers was prompted or seeded by the activist. Recommendations for improvements included enhancing the search function and providing hands-on training at the next community conference.

**Figure 2:**
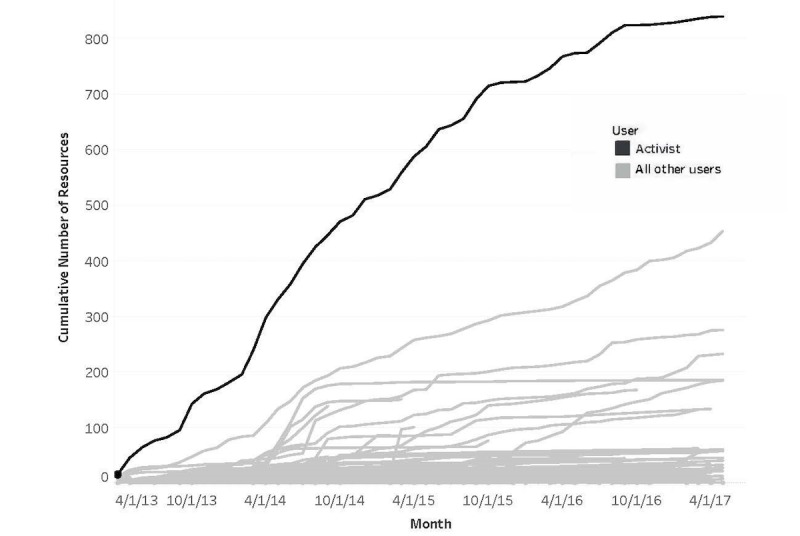
Activist user contributions compared to all other users over 4 years.

**Figure 3:**
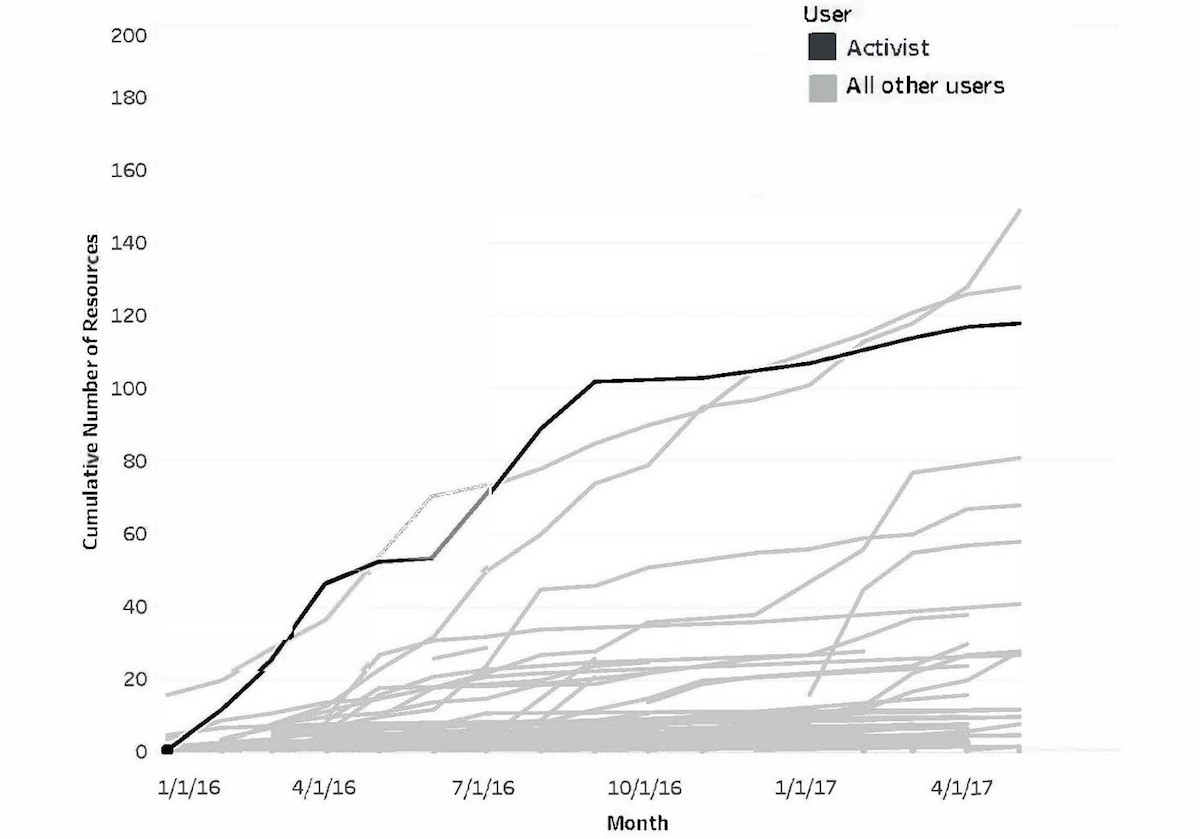
Contributions of some community members (emerging activists) approach and surpass contributions of the activist in final 15 months.

An indication that the activist was modeling behavior for the community was evident in that the cumulative activity of this individual exceeded that of all other users (Figure 2). While this role was critical to community building it did create a weak link, namely the person and the role were synonymous. A necessary feature of a sustainable community was the emergence of other users taking a similar role and being encouraged to do so by the activist. More recent behavior on the ICNExchange (Figure 3) showed that some other users have emerged as activists. Their cumulative behavior in the past year showed a pattern similar to and in some cases exceeding the activity level of the original activist.

**Figure 4:**
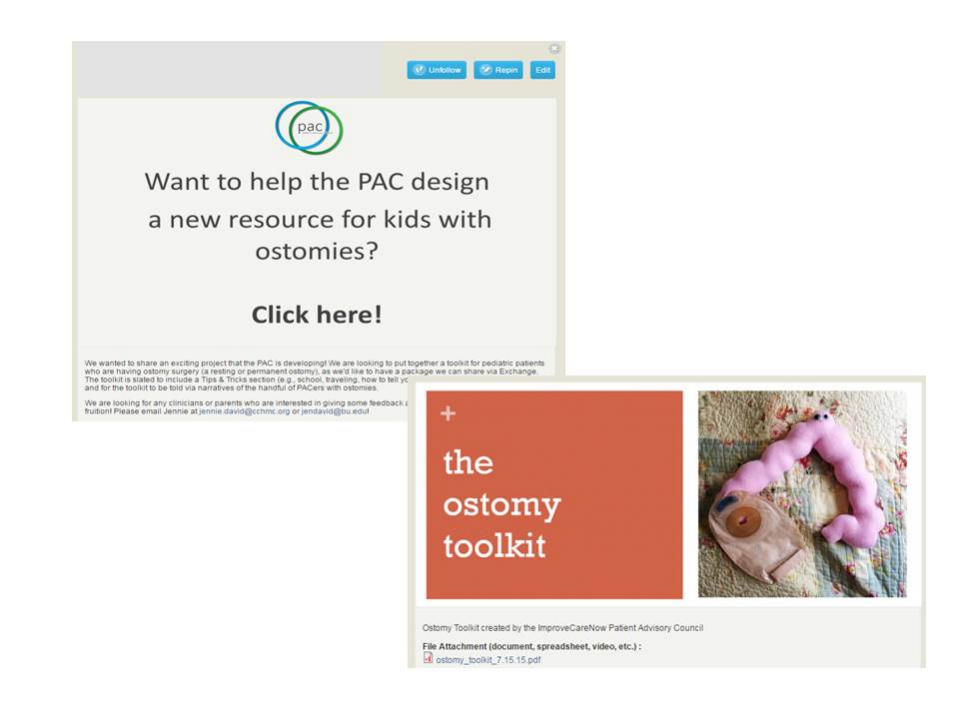
Pins illustrating the call to action and the resulting product, the ostomy toolkit.

Three key elements emerged as candidates for improvement in a next version. First, while the pin motif was familiar to the early users due to their familiarity with Pinterest, more recent feedback has indicated that the home page, with its many pins, can be confusing. Second, although the original vision of the Exchange was to enable collaborative creation of new resources, the capability of the Web platform is not yet ideal for collaborative cocreation. The ICNExchange has been used as a vehicle to announce a call to action and then post the finished product. For example, the patient advisory committee used the ICNExchange to invite the community to develop a new resource for kids with ostomies and later to be a distribution channel for the work (Figure 4) [33]. However, the work to cocreate the resource took place outside of the Exchange. Third, we have heard from our users that the search function needs improvement, specifically the ability to search across multiple data fields (eg, author and full text of attached documents).

The types of activities summarized in Table 2 suggest that the ICNExchange was useful in its original aim of facilitating the management and exchange of knowledge.

As the ICNExchange was used more, further utility was uncovered. One such use emerged from formal inquiry at the semiannual face-to-face meetings and led to making the distribution of meeting materials a function of the ICNExchange. Using the ICNExchange in this way was more efficient and reliable and reduced work for administrative staff. Adding Wi-Fi connectivity to in-person conferences allowed each user to access content on a laptop or tablet. As a result, the ICNExchange became a prominent feature of the network, and driving traffic to the commons increased the potential of further engaging users in making the Learning Exchange part of their individual work processes.

An additional enhancement made post release was the development of a visible database of the improvement goals established by care centers. The data entry and viewing functionality developed on the ICNExchange provides an opportunity for all care centers to see and share their goals and progress and identify those working to tackle similar issues.

## Discussion

### Principal Findings

The ICNExchange is a collaborative knowledge-sharing platform that allows members of an extended Learning Network to communicate and innovate across the globe. The various members of the community use it to share seamlessly, and it has extended the community’s all teach/all learn focus. The ICNExchange was designed and developed to be used by an ongoing community involved in improving outcomes for patients. The growth in active users and activities shows that it was acceptable as a first version of such a platform, use patterns and feedback show that it is feasible with room for improvement, and activity on the platform as well as additional uses attest to its utility.

Opportunities exist to continue the community-focused design process to meet the network’s evolving needs. Barriers to use, such as lack of time, perceived irrelevance, and information overload, as well as facilitators, such as a community of practice, training, and a community facilitator, are similar to those found in the literature [10]. New ideas for using the ICNExchange emerged over time as the community learned more about its use and capabilities and the design team remained engaged in monitoring acceptability, feasibility, and utility. This illustrates the key benefits of a flexible platform and ongoing design process that remains community- versus technology-focused. These are facilitators not readily apparent in other literature [10].

In such a large network, keeping participants connected is daunting as is ensuring the best tools and ideas are shared equally. Before launching this innovation, tools, ideas, and knowledge generated by individual care centers were relegated to email attachments and file cabinet drawers. Centers connected on monthly webinars and twice yearly in-person meetings, but little asynchronous collaboration or peer mentoring was possible. Our vision for the ICNExchange is continued evolution toward better curation and organization of health-improving ideas and best practices, leading to faster spread across more centers. We have the community of improvers and the will to collaborate using the ICNExchange but need to further cultivate the commons so everyone can easily find like-minded people, the tools they need, and the knowledge to implement shared tools well. While the ICNExchange has been an improvement in this vibrant community, as with any technology, with use, limitations and opportunities for improvement become apparent.

### Looking to the Future

As we design the next version, our vision is for a home page that is less cluttered and can be personalized for the specific user; the pin motif will still exist but will be in the background. We also learned that the multiple steps needed to create a pin is considered a barrier. In a future version, we plan to reduce the steps needed to add a pin to a single step that only involves uploading a resource. We intend to improve search by enabling the search of content in attachments, something that is not currently possible, as an adjunct to searching by taxonomy and keyword. Our vision for a future Learning Exchange would support work inside of a community commons to develop new and needed resources. Collaborators can have one place for ideation, creation, storage, and version control so that the Learning Exchange facilitates the collaboration to create new assets, not just the sharing of assets.

### Limitations

We framed this work as the development of an intervention to address the challenges of connecting people and their knowledge across a large and growing network. We engaged with the community regularly to understand what was useful and what was not useful and be aware of emergent and unanticipated needs. While necessary and important, such inquiry was driven by efforts in design and development and not an overarching research strategy. While structured inquiry was undertaken through interview and survey methods, the value of this inquiry was limited by the fact that such inquiry was not undertaken more often. Additionally, we have noted the importance of community engagement and the crucial role of an activist to facilitate that engagement and have described those actions. While important, actions alone are not sufficient. As we noted, the activist was a respected member of the community and this phrasing implies psychosocial features such as mutual trust and understanding. These and similar concepts are not well documented here; future research should be directed to a better understanding of the psychosocial milieu that makes community engagement successful.

### Conclusion

The scalability of the ICNExchange as a model for collaboration and information sharing is dependent on both extending the cocreation capability of the platform and transferring the model to new communities. Our vision for a Learning Exchange as a platform where network participants, made up of parents, patients, clinicians, and QI professionals cocreate resources to solve clinical improvement challenges depends on developing, extending, and enabling the creative commons of our collaborative network. The ICNExchange is a dynamic repository of solutions for chronic care across a spectrum of different conditions. Seamless sharing, applied to community-developed approaches to previsit planning, population management, self-management support, and QI, is an enabling strategy to improve health care delivery to chronic care patients. The ICNExchange fosters unique collaborations by allowing diverse and distributed groups to interact and share in collaborative spaces and share across the broader community. The Learning Exchange platform is now primed for rapid dissemination and transfer to other chronic conditions.

## Abbreviations

BTS: breakthrough series
IBD: inflammatory bowel disease
ICN: ImproveCareNow
LPN: licensed practical nurse
NP: nurse practitioner
PA: physician assistant
QI: quality improvement
RN: registered nurse
